# Graphene–Silicon Device for Visible and Infrared Photodetection

**DOI:** 10.1021/acsami.1c12050

**Published:** 2021-09-28

**Authors:** Aniello Pelella, Alessandro Grillo, Enver Faella, Giuseppe Luongo, Mohammad Bagher Askari, Antonio Di Bartolomeo

**Affiliations:** † Department of Physics and Interdepartmental Centre NanoMates, University of Salerno, via Giovanni Paolo II, Fisciano, Salerno 84084, Italy; ‡ CNR-SPIN, via Giovanni Paolo II, Fisciano, Salerno 84084, Italy; § IHP-Microelectronics, Im Technologie Park 25, Frankfurt Oder 15236, Germany; ∥ Department of Physics, Faculty of Science, University of Guilan, 41335-1914 Rasht, Iran

**Keywords:** graphene, Schottky diode, Gr-Si junction, heterojunction, photodetector, responsivity, visible, infrared, quantum efficiency, noise equivalent power

## Abstract

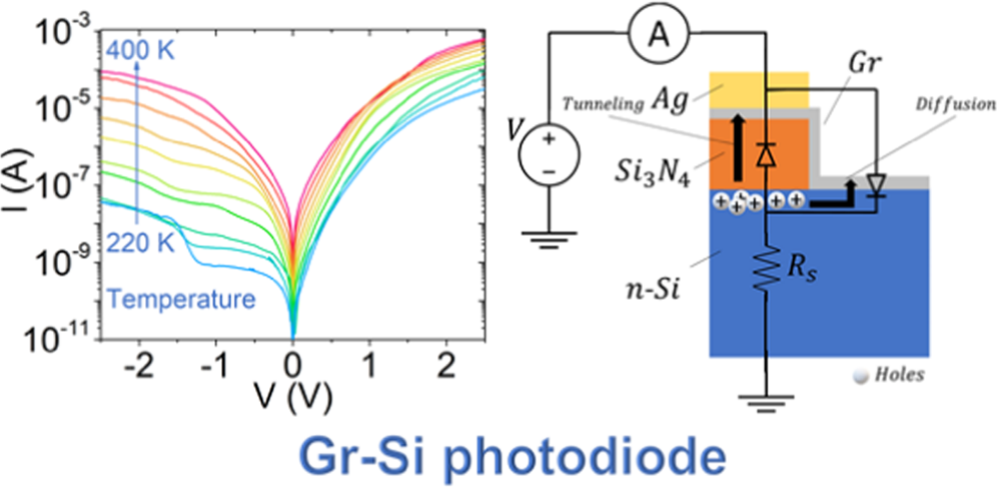

The fabrication of a graphene–silicon (Gr-Si) junction involves the formation of a parallel metal–insulator–semiconductor (MIS) structure, which is often disregarded but plays an important role in the optoelectronic properties of the device. In this work, the transfer of graphene onto a patterned n-type Si substrate, covered by Si_3_N_4_, produces a Gr-Si device, in which the parallel MIS consists of a Gr-Si_3_N_4_-Si structure surrounding the Gr-Si junction. The Gr-Si device exhibits rectifying behavior with a rectification ratio up to 10^4^. The investigation of its temperature behavior is necessary to accurately estimate the Schottky barrier height (SBH) at zero bias, φ_b0_ = 0.24 eV, the effective Richardson’s constant, A* = 7 × 10^–^^10^ AK^–2^ cm^–2^, and the diode ideality factor *n* = 2.66 of the Gr-Si junction. The device is operated as a photodetector in both photocurrent and photovoltage mode in the visible and infrared (IR) spectral regions. A responsivity of up to 350 mA/W and an external quantum efficiency (EQE) of up to 75% are achieved in the 500–1200 nm wavelength range. Decreases in responsivity to 0.4 mA/W and EQE to 0.03% are observed above 1200 nm, which is in the IR region beyond the silicon optical band gap, in which photoexcitation is driven by graphene. Finally, a model based on two parallel and opposite diodes, one for the Gr-Si junction and the other for the Gr-Si_3_N_4_-Si MIS structure, is proposed to explain the electrical behavior of the Gr-Si device.

## Introduction

Silicon has been leading the development of the semiconductor technology holding a dominant position in the microelectronics field for decades. However, silicon as an optoelectronic material suffers from the short bandwidth and the large surface reflectivity that limit the responsivity and the application of silicon-based photodetectors to near-infrared (NIR) bands. NIR photodetection, particularly at 1550 nm, is crucial for a variety of applications, ranging from optical communications^1−3^ to remote sensing.^4−6^

Graphene offers a very attractive platform for advanced optoelectronic applications due to its high conductivity, zero band gap, low noise, flexibility, chemical stability, and other extraordinary properties.^7−10^ Owing to its semimetal behavior and tunable energy Fermi level, it enables new functionalities in traditional electronic and optoelectronic devices.^11,12^ For instance, silicon can be joined to graphene to form a Schottky diode, which is used as a bias-controlled photodetector.^12−15^ Graphene can replace the metal contact of a Schottky junction and make shallow junctions with enhanced photoresponse.^16,17^ In reverse bias, photons with energy higher than the semiconductor band gap, absorbed in the semiconductor depletion layer, induce the formation of photocharges that are separated by the junction built-in field, originating a photocurrent. Moreover, photons with sub-band-gap energy can be adsorbed by graphene and inject electrons over the Gr-Si barrier, leading to a charge flow from the graphene to the semiconductor.^17,18^ Furthermore, the Gr-Si junction is a basic element for novel electronic devices for the integration of graphene into the existing semiconductor technology.^19−21^

A Gr-Si junction is fabricated on a Si substrate covered by a SiO_2_ dielectric layer, typically 100–300 nm thick or less. To increase the capacitance of the MIS structure, a thinner SiO_2_ layer down to 20 nm has been used.^22^ The etching of a window in the SiO_2_ cap layer exposes the Si area for the formation of the Gr-Si junction. The transferred graphene covers the bare Si area and encroaches upon the oxide layer for the formation of contacts with metal leads. Such an encroachment of graphene over SiO_2_ originates a MIS structure, namely, a Gr-SiO_2_-Si structure, which is in parallel with the Gr-Si junction. It has been shown that the MIS structure affects the current–voltage (*IV*) and capacitance–voltage characteristics of the junction^23^ and enhances its photodetection capability.^24−26^ The important role of the MIS structure has been pointed out by Riazimehr et al.,^25,27^ yet it is still neglected in several studies. In this work, we further point out that the MIS structure is essential to understand the electronic and optoelectronic properties of Gr-Si devices.

We transfer graphene monolayers, produced by chemical vapor deposition (CVD), onto a n-Si wafer covered by a patterned Si_3_N_4_ layer that replaces the traditional SiO_2_ dielectric. Si_3_N_4_ films are excellent diffusion barriers (for metal, water, oxygen, etc.) and have high dielectric constant (∼7.5) and dielectric strength (>10^7^ V/cm). In such a way, we form Gr-Si junctions in parallel with Gr-Si_3_N_4_-Si MIS structures. The original use of Si_3_N_4_ instead of SiO_2_ allows us to enhance the MIS capacitance by increasing the dielectric constant and reducing the insulator thickness to 15 nm. We highlight the importance of the Gr-Si_3_N_4_-Si MIS capacitor as part of the Gr-Si device. The MIS capacitor plays a significant role in improving the optical and electronic properties of Gr-Si photodiodes, as reported in recent studies.^25,27−29^ Our special layout leads to the observation of unreported features, namely, a kink in the reverse-bias *IV* characteristics, which we attribute to Fowler–Nordheim (FN) tunneling and model as two parallel and opposite diodes. We demonstrate that the Gr-Si_3_N_4_-Si structure is a booster for the photoresponse as it becomes the reservoir of photogenerated holes that contribute to the photocurrent in reverse bias. Photogenerated holes accumulated at the Si_3_N_4_-Si interface that tunnel through the Si_3_N_4_ by the FN mechanism increase the photocurrent and enhance the responsivity of the device.

We evaluate transport parameters such as Gr-Si SBH and ideality factor, and we study the responsivity of the device in the visible and near-infrared regions demonstrating a promising photodetector. We show that the investigation of the temperature behavior of the Gr/Si device is necessary to accurately evaluate the SBH at zero bias and that the approach based on a single room-temperature *I*–*V* curve, although very common, leads to a substantial overestimation of the barrier height.

Our results can be adapted to other devices such as gated two-dimensional (2D)–2D heterojunctions or transparent conductive materials on three-dimensional (3D) semiconductors.

## Materials and Methods

Samples were prepared on doped n-Si (100) wafers with a resistivity of ∼10 Ωcm, corresponding to a phosphorus dopant density of ∼4.5 × 10^14^ cm^–3^. A 15 nm thick silicon nitride (Si_3_N_4_) was deposited by CVD. Then, a 3 × 3 mm^2^ trench was patterned by lithography and wet etching of the silicon nitride. The trench area was further cleaned by hydrofluoric acid immediately before the Gr deposition to prevent or limit the formation of native oxide.

An ∼5 × 7 mm^2^ Gr sheet was transferred onto the Si substrates by a wet method (the details are reported elsewhere^30^) to cover the Si trench while extending over the surrounding Si_3_N_4_ layer, thus acting both as the anode of the Gr-Si junction and the top (gate) electrode of the Gr-Si_3_N_4_-Si MIS structure. Contacts to graphene were realized by Ag paste of about 1 mm diameter. The achievement of low-resistance ohmic contacts with graphene^31^ was verified by measuring linear *IV* characteristics between Ag-Gr contacts in two-probe configuration. Likewise, the Ag paste was spread on the scratched backside of the Si substrate to guarantee an ohmic back contact.

**Figure 1:**
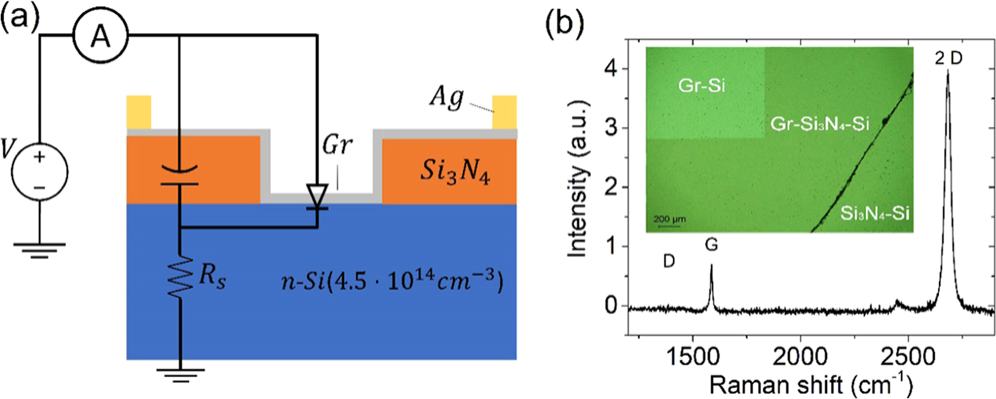
(a) Schematic of the device showing a Gr-Si junction modeled by a diode in parallel with a MIS structure, here represented as a capacitor. (b) Raman spectrum confirming high-quality monolayer graphene. The inset shows an optical top image of the device, displaying the graphene flake and the window etched through the Si_3_N_4_ layer corresponding to the Gr-Si junction.

The schematic of the device is reported in Figure 1a. The Raman spectrum (Figure 1b) provides a clear evidence of a high-quality monolayer Gr layer, confirmed by the high-2D/G-intensity peak ratio and the negligible defect-related D-peak (∼1350 cm^–1^).

## Results and Discussion

**Figure 2:**
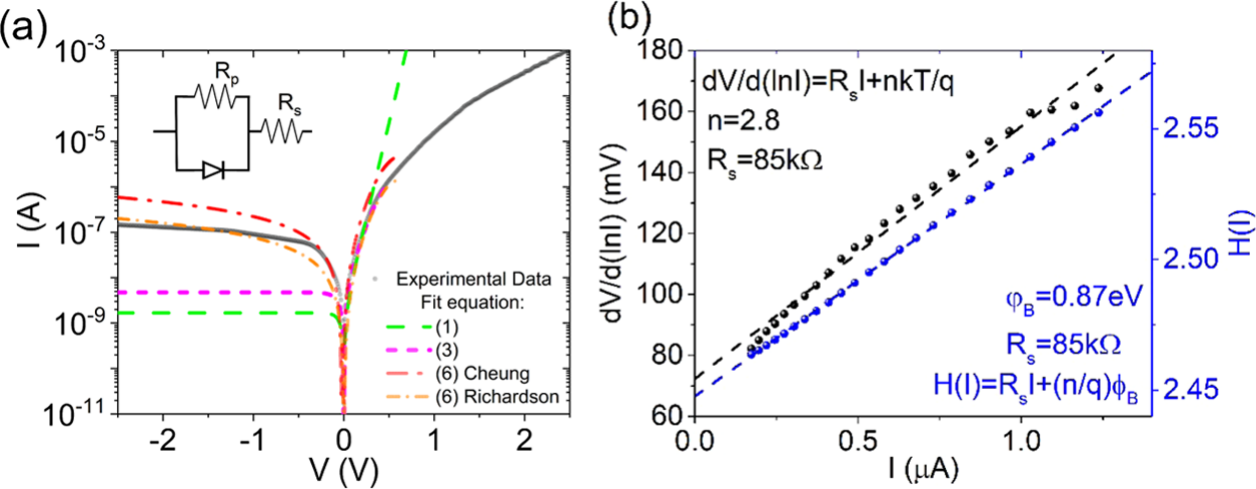
(a) Measured *IV* characteristic of the device in the dark (black line). The green and magenta lines represent the fit using, respectively, eqs 1 and 3. Red and orange lines represent the fit using eq 6 with Cheung and Richardson’s parameters, respectively. The inset represents the diode model with a series and a parallel resistance corresponding to eq 6. (b) Cheung’s method plots for the evaluation of Schottky barrier height, ideality factor, and series resistance.

Figure 2a shows the semilogarithmic plot of the *IV* characteristic of the Gr-Si device in the dark at 300 K and atmospheric pressure. The device exhibits a rectifying behavior with rectification ratio ∼10^4^ at *V* = ±2.5 V and shape that suggests the use of the diode equation for the current
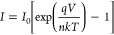
1where *I*_0_ is the reverse saturation current, *q* is the electron charge, *n* is the ideality factor, *k* is the Boltzmann constant, and *T* is the temperature. The ideality factor considers deviations from pure thermoelectric transport (*n* = 1), which can take place in a Schottky diode. The presence of defects or unwanted insulating layers can cause Schottky barrier inhomogeneities and increase the ideality factor.^32−34^ The green dashed line in Figure 2a represents the fit to the experimental data of eq 1 with *I*_0_ and *n* as fitting parameters, obtained in the forward region 0 < *V* < 0.4 V. Indeed, in this region, any series resistance can be neglected, and the best fit is obtained with *n* = 2.02 and *I*_0_ = 1.69 × 10^–9^ A. Using the estimated value of *I*_0_ and referring to the thermionic theory
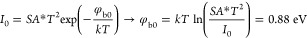
2where *S* = 0.09 cm^2^ is the area of the junction, A* = 112 cm^–2^ K^–2^ is the Richardson constant for n-Si, and φ_b0_ is the SBH at zero voltage. This estimation is affected by an error, which is related to the arbitrary choice of the forward region for the fit and might contain a huge systematic error due to the assumed values of *S* and A* (see below).

A more realistic model includes a series resistance *R*_s_. We then follow Cheung’s method^35^ for evaluating the diode parameters. According to Cheung’s method, eq 1 with a series resistance becomes
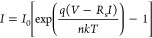
3which, for *V* – *R*_s_*I* ≫ *nkT*/*q*, provides
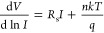
4From the fit of eq 3, we can evaluate *R*_s_ and *n*, which can be used to estimate φ_b0_, defining the following function
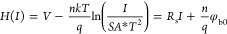
5Figure 2b shows the plots of  and *H*(*I*) as a function of *I* from which we extract the following parameters: *n* = 2.8, *R*_s_ = 85 kΩ, and φ_b0_ = 0.87 eV. Using the so-estimated *I*_0_, *R*_s_, and *n* in eq 3, we obtain the magenta dotted curve shown in Figure 2a. Such a curve provides a good fit to the forward current until the flat band condition is reached (*V* – *R*_s_*I* ≃ 0, which occurs at *V* ≃ 0.59 V), where the diode equation does not apply anymore.

As all fittings fail in the reverse region, we further improve the model by considering a parallel resistance *R*_p_, which takes into account possible leakages through the dielectric or the substrate edge^24^
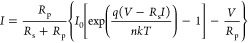
6Equation 6 provides an acceptable fit both in reverse and forward biases, as shown by the red dash-dot line in Figure 2a, when *R*_p_ = 4 MΩ and *I*_0_ = 2.3 ×10^–8^ A.

**Figure 3:**
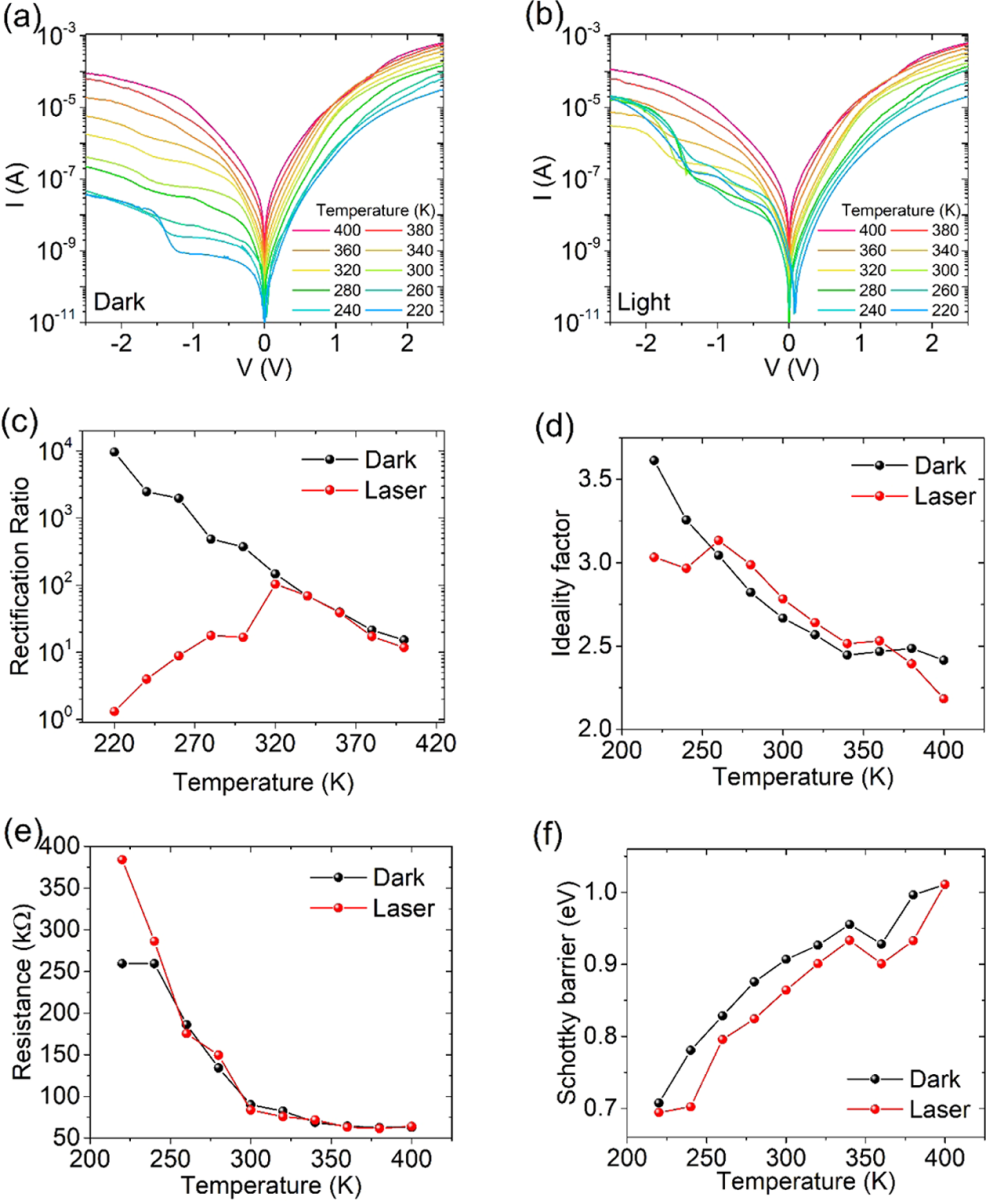
*IV* characteristics versus temperature ranging from 400 to 220 K (a) in the dark and (b) under light (3 mm diameter spot, incident power 1 mW/cm^2^, wavelength λ = 500 nm). (c) Rectification ratio at *V* = ±2.5 V, (d) ideality factor, (e) series resistance, and (f) Schottky barrier height versus temperature, estimated using Cheung’s method.

Figure 3a,b displays the *IV* characteristics of the Gr-Si device at different temperatures, from *T* = 400 to 220 K, in the dark and under illumination by a supercontinuum laser with a 3 mm diameter spot (incident power 1 mW/cm^2^, wavelength λ = 500 nm), respectively. The plots show that lowering the temperature suppresses both the forward and reverse currents, as predicted by the thermionic theory (eqs 1 and 2). Noteworthy is a substantial difference between the current in the dark and under illumination is observed only at temperatures below 320 K and in reverse bias. Light does not appreciably change the forward current and, at higher temperatures, the reverse current. At low temperatures, the *IV* curves present sudden curvature changes (one or more kinks) in reverse bias probably caused by the parallel MIS capacitor, as explained below.

Figure 3c shows the rectification ratio at *V* = ±2.5 V. In the dark, the rectification ratio increases with decreasing temperature, indicating that the Schottky barrier becomes more efficient in suppressing the electron flow from graphene to silicon (reverse current) when the temperature is lowered. Under illumination, the rectification ratio overlaps that measured in the dark for temperatures higher than 320 K, i.e., when thermal generation overcomes photogeneration. Below 320 K, the rectification ratio decreases with decreasing temperature because the reverse current becomes more and more dominated by the photogeneration. This result indicates that the suppressed dark (reverse) current at a low temperature is favorable to photodetection. Indeed, at low temperatures, the reverse current is substantially enhanced by the electron–hole photogeneration.

From Cheung’s method, we obtained the values of *n*, *R*_s_, and φ_b0_ as a function of temperature, respectively, reported in Figure 3d–f, in the dark (black dot-lines) and under light (red dot-lines). The temperature dependence of the ideality factor in Figure 3d shows a descending behavior for increasing temperature. This feature indicates that deviations from the ideal thermionic behavior of the diode are more effective at lower temperatures when thermionic emission is suppressed, and tunneling and diffusion might become comparatively relevant. The dependence on the temperature of the series resistance is typical of a semiconductor and is probably dominated by the silicon substrate.

Finally, Figure 3f shows that the SBH increases with increasing temperature. This is a well-known effect when there is barrier inhomogeneity.^36−38^ At low temperatures, the reduced thermal energy makes the carrier cross the barrier mainly in the position where the SBH is lower, thus resulting in a reduced SBH.

**Figure 4:**
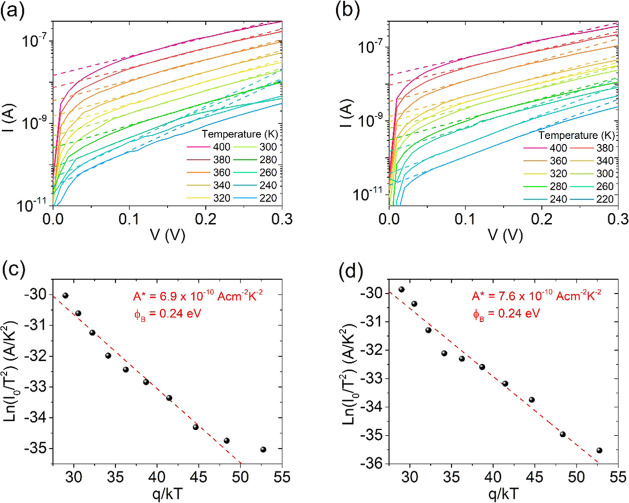
Linear fits used to extract *I*_0_ at *V* = 0 V (a) in the dark and (b) under illumination light (3 mm diameter spot, incident power 1 mW/cm^2^, wavelength λ = 500 nm). Richardson’s plots obtained from *IV* measurements (c) in the dark and (d) under illumination.

The obtained zero-bias SBH, φ_b0_ = 0.87 eV, although consistent with some previous works,^39,40^ exceeds the prediction of the Schottky–Mott model, φ_b0_ = Φ_g_ – χ_Si_ = 0.5 eV (Φ_g_ = 4.54 eV is the commonly used work function of graphene and χ_Si_ = 4.05 eV is the electronic affinity of Si) and should result in lower reverse current and higher rectification ratio than the ones we observed. We note that a large discrepancy can be found in the literature for the Gr-Si SBH (from 0.2 to 0.9 eV^24,39−45^) estimated from electrical transport and that complementary techniques such as X-ray photoemission spectroscopy lead to much lower barrier height.^43^ Furthermore, several experimental studies of the Gr-Si Schottky junction have indicated that an effective Richardson constant, order of magnitude lower than 112 A cm^–2^ K^–2^, is needed to account for the experimental data.^21,23,36,37,46,47^ Therefore, to make an estimation of the SBH independent of the effective Richardson constant A* and to avoid relying on a single *IV* curve, we extracted the SBH and, as byproduct, the effective Richardson constant from the *IV* characteristics measured at different temperatures (Figure 3a,b). The linear fittings of the forward current were used to extrapolate the current *I*_0_ at *V* = 0 V, as shown in Figure 4a,b. According to eq 2, φ_b0_ and A* can be obtained from the slope and intercept of ln (*I*_0_/*T*^2^) versus  (Richardson’s plot in Figure 4c,d). The estimated A* ≈ 7 × 10^–10^ A cm^–2^ K^–2^ is significantly lower than the theoretical one (112 A cm^–2^ K^–2^) used for previous calculations, and the obtained SBH reduces to φ_b0_ = 0.24 eV, consistent with the modest rectification ratio and the results from similar devices.^21,23,36,41,48,49^ Although the origin of a lower effective Richardson constant is still under debate,^18,50^ the inadvertent presence of a native oxide layer at Gr-Si interface,^24^ the massless Dirac fermion nature of carriers in graphene,^13^ and a Landauer transport mechanism^46^ have been invoked as an explanation. We point out that the SBH does not change when measured in the dark or under illumination. This is due to the negligible effects of light on the forward *IV* characteristics of the device. The *I*_0_ value obtained by eq 2 with φ_b0_ and A* from Richardson’s plot, inserted in eq 6, provide the best fit to the data, as shown by orange dash-dot line in Figure 2a (the other fitting parameters are *R*_p_ = 12 MΩ, *I*_0_ = 5.85 × 10^–9^ A). The excellent agreement between experimental data and model is taken as a confirmation of the higher accuracy of the Richardson plot-based method.

**Figure 5:**
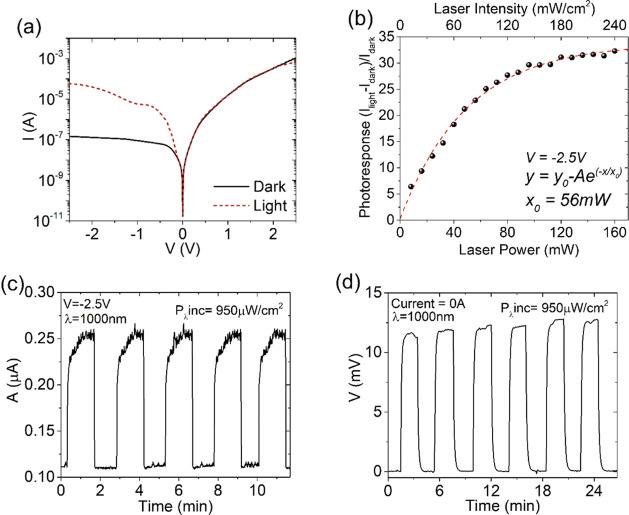
(a) *IV* characteristics in the dark and with incident white laser. (b) Photoresponse as a function of the laser emitted power and the laser integral intensity incident on the device (in mW/cm^2^). (c) Photocurrent when the photodetector is operated in the photocurrent mode at *V* = −2.5 V and (d) photovoltage mode at *I* = 0 A under a laser beam with 1000 nm wavelength and 950 μW/cm^2^ light intensity on the device.

To further investigate the physical mechanisms of charge transport, we tested the optical response of the device. Figure 5a shows the *IV* characteristics in the dark and under illumination by a supercontinuum white laser. Tuning the emission power of the laser from 0 to 180 mW (corresponding to the integral intensity of 0–240 mW/cm^2^ on the device and considering a light loss factor of 85% in our measurement setup over the entire spectral range), we obtained the photoresponse of the device, defined as (*I*_light_ – *I*_dark_)/*I*_dark_ at *V* = −2.5 V, as shown in Figure 5b. The data follow an exponential law with the e-folding factor *x*_0_ = 56 mW, which ensures that a maximum light effect is reached above ∼170 mW with unfiltered laser light. Accordingly, the laser was set to a maximum power of 180 mW for further tests of the Gr-Si device in photocurrent and photovoltage mode.

Setting the voltage to *V* = −2.5 V, we performed a series of measurements exposing it to the laser beam with wavelength emission of λ = 1000 nm, as representative of NIR light above the silicon band gap. The device reacts to the laser with fast and repeatable photocurrent (Figure 5c). Furthermore, at zero current, we also observed a photovoltaic effect as reported in Figure 5d, where a voltage of about 12 mV appears at the electrode in response to a laser pulse. Due to the low incident power (950 μW/cm^2^), possible thermal effects can be excluded. Figure 5c,d shows that under illumination, the device generates both a current and a voltage, i.e., it can be operated in a self-powered mode, consistent with other works in the literature.^51−53^

**Figure 6:**
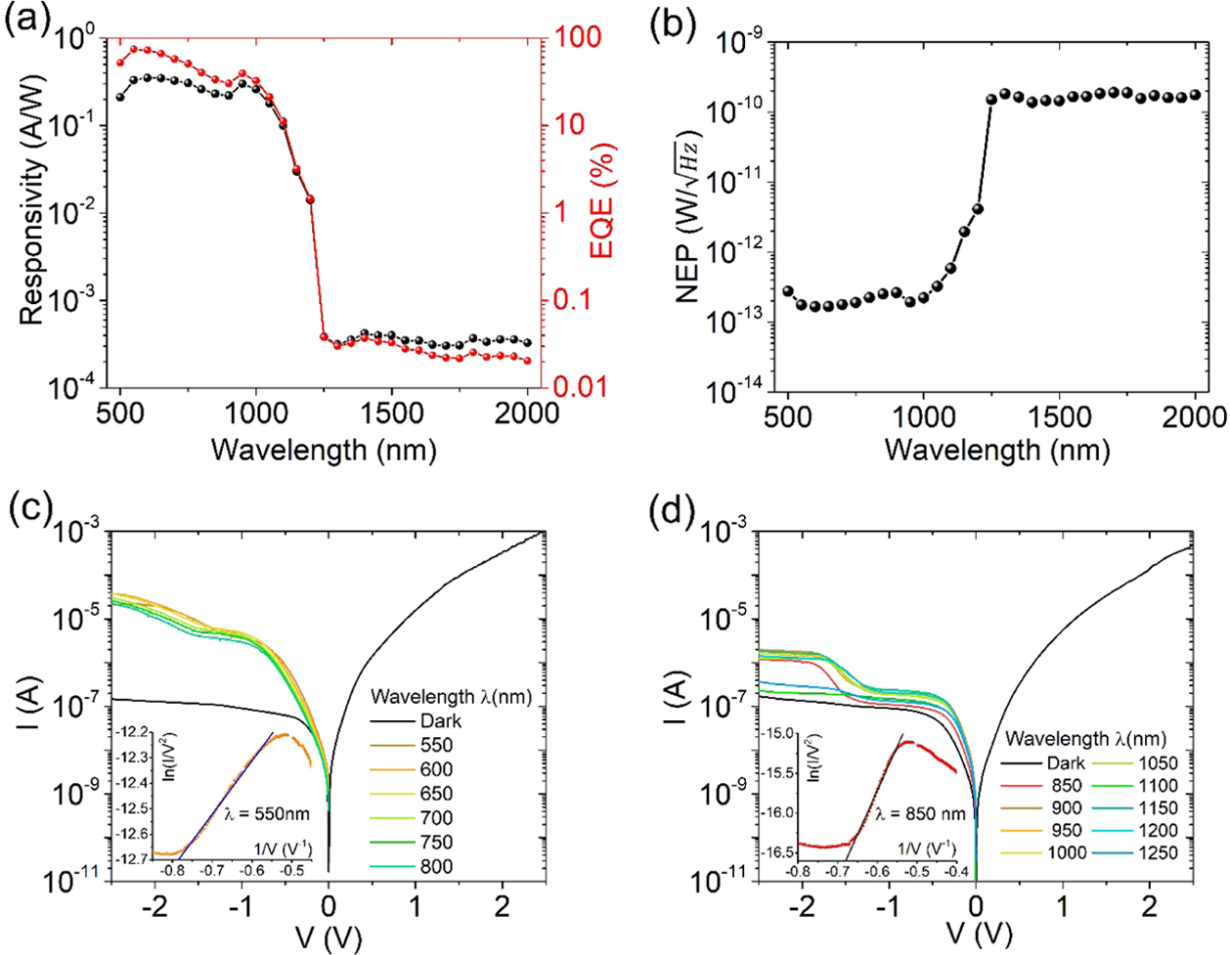
(a) Responsivity and EQE of the device in the visible and IR spectral regions. (b) NEP and (c) IV characteristics in the dark and under light at different wavelengths in the (c) visible and (d) near-spectral IR regions. The insets in (c) and (d) show the Fowler–Nordheim plots of the reverse IV characteristic at 550 and 850 nm, respectively.

To complete the optoelectronic characterization of the device, we investigated the spectral response in the 500–2000 nm wavelength range by sampling the spectrum of the supercontinuum laser in intervals of 50 and 20 nm bandwidth. Figure 6a reports the responsivity of the device, defined as the ratio of photocurrent to the incident power, *R* = (*I*_light_ – *I*_dark_)/*P*_inc_(λ) along with external quantum efficiency  (λ is the wavelength, *h* is Planck’s constant, and *c* is the speed of light). It shows an EQE around 75% for λ < 1100 nm, i.e., when photoconversion occurs mainly in Si, followed by a sudden drop to 0.03% when the energy of the incident light is below the band gap of Si. For λ > 1100 nm, photoexcitation occurs mainly in graphene and the EQE, as reported elsewhere.^9,54,55^ We highlight that the obtained external quantum efficiency is consistent with the highest value reported in the literature over the investigated spectral range.^22,53,56−60^

As an additional figure of merit, Figure 6b shows the noise equivalent power  that indicates the minimum detectable power. Obviously, the higher quantum efficiency corresponds to the lower detection power.

Figure 6c,d shows the *IV* characteristics under light at different wavelengths. It can be observed that a kink forms at about *V* = −1.2 V in the reverse curves. The photocurrent at a given illumination and wavelength reaches a plateau at a high reverse bias when it is limited by the photocarrier generation rate.

**Figure 7:**
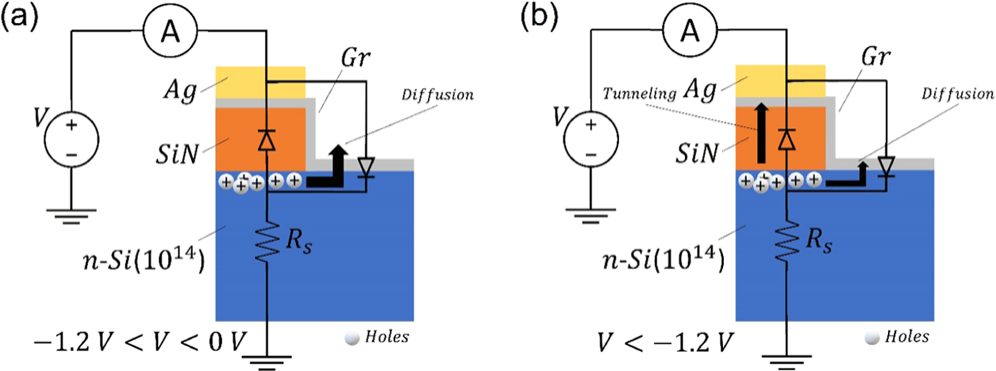
Schematic model of the Gr-Si device and charge carrier transport in reverse bias for (a) −1.2 V < *V* < 0 V and (b) V < −1.2 V.

The observed optoelectronic behavior of the Gr-Si device can be understood by considering the parallel Gr-Si_3_N_4_-n-Si structure, which in forward behaves like a MIS capacitor charged by an electron on the Si side. Such an electron can diffuse to the Gr-n-Si junction and contribute to the forward current. In reverse bias, the negative voltage attracts holes at the Si-Si_3_N_4_ interface. As the holes accumulate, the Si undergoes an inversion and becomes p-type. When the voltage is high enough to enable tunneling through the insulator, a p-type Schottky diode is formed in the MIS region. This means that, in reverse bias, the device behaves as two parallel and opposite diodes, a reverse Schottky diode due to the Gr-Si junction and a forward MIS diode formed by the Gr-Si_3_N_4_-p-Si structure. This parallel configuration explains the kinks at about *V* = −1.2 V. Indeed, for −1.2 V < *V* < 0 V, holes accumulated at the interface Si-Si_3_N_4_ can only diffuse toward the Gr-Si junction and contribute to its reverse current (Figure 7a), originating the leakage of ∼10^–7^ A. For *V* < −1.2 V, the electric field enables also FN tunneling^61,62^ through the Si_3_N_4_ layer (Figure 7b), resulting in an increase of current, which generates the aforementioned kinks. A current plateau is reached at a high reverse voltage because of the thermal or photogeneration-limited rate in Si. The FN tunneling mechanism is confirmed by the FN analysis of the reverse IV characteristics shown as insets in Figure 6c,d. Indeed, the linear behavior of the FN plot over the range *–*0.8 V^–1^ < V^–1^ < −0.5 V^–1^ (corresponding to −2.0 V to −1.25 V) demonstrates that FN tunneling occurs below the kink voltage.

## Conclusions

We have studied a Schottky Gr-Si junction in parallel with a Gr-Si_3_N_4_-Si structure, forming a composite Gr-Si device with high responsivity and external quantum efficiency in the visible and NIR regions. The device can be operated in both photocurrent and photovoltage modes. We have evaluated the relevant parameters of the junction and shown that only a detailed *IV*–*T* analysis leads to a realistic estimation of the Gr-Si Schottky barrier parameters. We have detected the appearance of a kink in the reverse current, and we have proposed a model based on two parallel back-to-back diodes to explain it. We have clarified how the parallel Gr-Si_3_N_4_-Si structure introduced in the fabrication of the device contributes to the optoelectronic properties of the Gr/Si heterostructure.
